# Improving generation length estimates for the IUCN Red List

**DOI:** 10.1371/journal.pone.0191770

**Published:** 2018-01-25

**Authors:** Robert S. C. Cooke, Tania C. Gilbert, Philip Riordan, David Mallon

**Affiliations:** 1 Marwell Wildlife, Thompson’s Lane, Colden Common, Winchester, Hampshire, United Kingdom; 2 University of Southampton, University Road, Southampton, Hampshire, United Kingdom; 3 Division of Biology and Conservation Ecology, Manchester Metropolitan University, Manchester, Greater Manchester, United Kingdom; Universita degli Studi di Napoli Federico II, ITALY

## Abstract

The International Union for the Conservation of Nature (IUCN) Red List classifies species according to their risk of extinction, informing local to global conservation decisions. Here we look to advance the estimation of generation length, which is used as a time-scalar in the Red List as a way of accounting for differences in species’ life-histories. We calculated or predicted generation length for 86 species of antelope following the *Rspan* approach. We also tested the importance of both allometry (body-mass) and phylogeny (phylogenetic eigenvectors) as predictors of generation length within a Phylogenetic Eigenvector Map (PEM) framework. We then evaluated the predictive power of this PEM and two binning approaches, following a leave-one-out cross-validation routine. We showed that captive and wild longevity data are nonequivalent and that both body-mass and phylogeny are important predictors for generation length (body-mass explained 64% and phylogeny 36% of the partitioned explained variance). Plus, both the PEM, and the binning approach that included both taxonomic rank and body-mass, had good predictive power and therefore are suitable for extrapolating generation length to missing-data species. Therefore, based on our findings, we advise separating captive and wild data when estimating generation length, and considering the implications of wild and captive data more widely in life-history analyses. We also recommend that body-mass and phylogeny should be used in combination, preferably under a PEM framework (as it was less reliant on available reference species and more explicitly accounts for phylogenetic relatedness) or a binning approach if a PEM is not feasible, to extrapolate generation length to missing-data species. Overall, we provide a transparent, consistent and transferable workflow for improving the use of the *Rspan* method to calculate generation length for the IUCN Red List.

## Introduction

Understanding species’ vulnerability to extinction, in order to mitigate impacts and prevent the loss of biodiversity, is a major goal of conservation biology [1]. The IUCN Red List of Threatened Species, hereafter referred to as the Red List, has become widely recognized as the most comprehensive and accepted authority on the conservation status of species [2,3]. The Red List has been implemented in scientific, political and popular contexts as a means of highlighting the world’s most threatened species and directing conservation actions [4], with over 85,000 species assessed; as well as for reporting on biodiversity trends through the Red List Index [5].

Through an established assessment process, the IUCN protocol classifies species into different categories of risk (ranging from Least Concern to Extinct) using a formal set of five criteria supported by quantitative thresholds [6]. The Red List process is then applied by qualified assessors, using the best available data, which is often voluntarily provided by species experts [4,7]. However data limitations can sometimes result in inconsistencies and inaccuracies [4,7]. The Red List is therefore dependent on adequate and accurate information on wild populations (which are the only ones eligible for assessment on the Red List) [6]. Whilst efforts are made to validate assessments through a rigorous review process, inaccuracies can confound reporting against international targets, such as the Aichi 2020 biodiversity targets set by the Convention on Biological Diversity, as well as misdirecting scarce conservation funding [7].

Much research effort has been targeted towards improving the accuracy of the population and geographic range data that inform the Red List. However, less focus has been paid to the time scalar against which the criteria are assessed [8]. For instance, three of the five criteria (Criteria A, C1, and E) use time-scales measured as the longer of either a fixed time period or a number of generation lengths [4]. The generation length time horizon is implemented to account for variation in life-histories among species, such as the different rates at which taxa survive and reproduce [9]. However inaccurate estimates of generation length can result in population declines being measured over an incorrect time period, potentially leading to false estimates of extinction risk [8]. Thus, the ability to generate accurate estimates of generation length across species with high reliability is clearly important for the assessment process, and by extension, for conservation prioritization [1,8].

Several methods for calculating generation length are available [6,9], where generation length is defined as: “the average age of parents of the current cohort (i.e., newborn individuals in the population). Generation length therefore reflects the turnover rate of breeding individuals in a population” [9]. This can be interpreted as either the mean age at which a cohort of individuals produce offspring; the age at which an individual achieves half of its total reproductive output; the mean age of parents in a population at the stable age distribution; or the time required for the population to increase by the replacement rate [9].

In the case of the 2008 Red List Assessment for antelope implemented by the IUCN SSC Antelope Specialist Group, expert-based generation length estimates were used. These were based on one of the methods detailed by the IUCN Standards and Petitions Subcommittee [9] (*Rspan*; see below) using life-history and demographic data for those species that were available at the time and extrapolating the resulting generation length estimates to other species, based on general patterns of taxonomic relatedness (e.g., genus, family) and body size (e.g., small, medium, large). In several cases, data on age at last reproduction (maximum longevity used as a proxy) were available only from captive animals. Since longevity is almost always expected to be shorter under wild conditions, an arbitrary estimate of 75% of the captive figure for longevity was used. Whilst providing a useable proxy for actual generation lengths, the limitations and assumptions of this approach are acknowledged. However with increasing availability of life-history data and statistical techniques we now have the opportunity to empirically calculate and extrapolate generation length estimates for multiple species.

Missing values are commonplace in life-history trait databases. Yet the Red List requires an estimate of generation length for every species assessed using Criteria A, C1, or E [6]; therefore the commonly implemented data deletion approach (removing species with incomplete data) is not applicable. The guidance for missing-data species has always been to extrapolate generation length from closely related taxa [9]. We set out to establish a more robust way of estimating missing data by investigating the importance of both phylogenetic and allometric factors as predictors of generation length, due to their expected evolutionary and physiological influence respectively. For instance, most species traits exhibit some degree of allometric scaling [10]. In addition, for any group of phylogenetically clustered taxa, phenotypic similarities will not be independent, reflecting a mixture of convergent evolutionary responses to a similar underlying causes and limited divergence from a shared common ancestor; therefore evolutionary history should be explicitly considered [11].

Here we aim to improve the accuracy of generation length estimates by addressing potential issues across the methodological workflow. From potential biases in the underlying data, in particular the use of both captive and wild data, to identifying the best predictive framework and predictor variables to extrapolate generation length to missing-data species. Using antelopes as an example, we demonstrate that estimates for generation length can be considerably improved using our approach.

## Materials and methods

### Focal species

**Fig 1:**
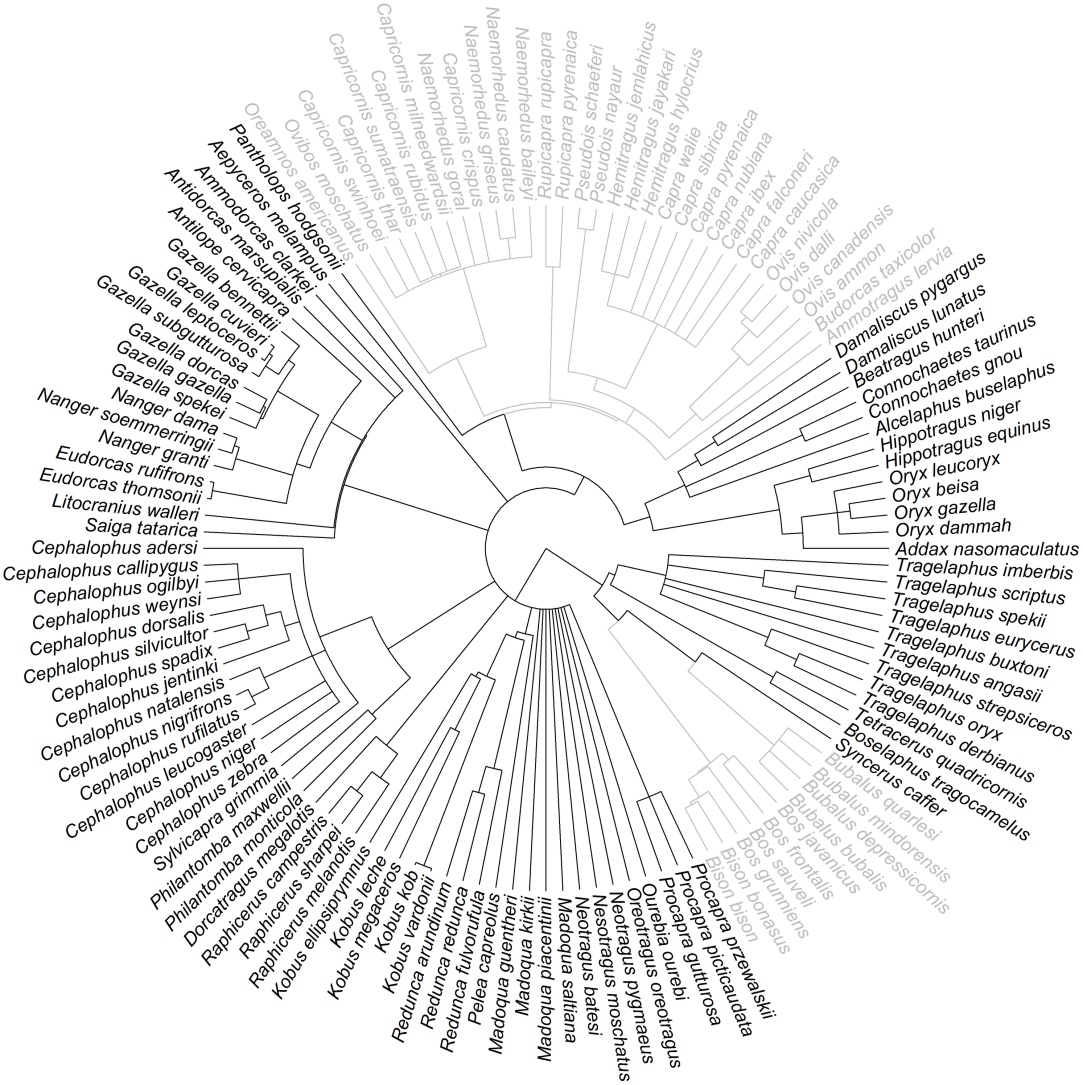
Phylogenetic tree of Bovidae (128 species), based on an updated supertree of extant mammals [12]. Those species included in our models are marked in black (86 species). Additional analyses were also performed excluding chiru (*Pantholops hodgsonii*) and African buffalo (*Syncerus caffer*) (S3 Appendix).

We focused on 86 extant Bovidae species from 34 genera that fall within the IUCN SSC Antelope Specialist Group’s remit (generally termed antelopes; Fig 1). Additional analyses were also performed excluding chiru (*Pantholops hodgsonii*), which are more closely related to Caprinae and African buffalo (*Syncerus caffer*), which are taxonomically close to wild cattle (S3 Appendix).

### Estimating generation length

Generation lengths are based on empirical estimates of life-history traits, with the IUCN recommending three techniques: 1) life-table method; 2) mortality method; and 3) reproductive lifespan (*Rspan*) method [9]. The applicability of each approach varies depending on data resources and availability. The life-table method is the preferred technique of calculating generation length for the Red List [9]: *Generation length = Σxl*_*x*_*m*_*x*_*/Σl*_*x*_*m*_*x*_ where x is age from zero to the last age of reproduction; *m*_*x*_ is the fecundity at age *x*; and *l*_*x*_ is survivorship up to age *x* [9]. However this is only applicable to species where life-table information exists, which is often not the case for wild populations, though such data may be available for captive populations. We had robust life-table data for very few species, all of which were from captive populations and thus suffer from additional biases, such as management intensity and manipulated breeding (often an aim of captive management is to extend generation length to reduce loss of genetic diversity). Thus the life-table method was not implemented even for comparative purposes. Therefore, when these data are lacking, approximations or extrapolations are needed. For vertebrates these include the mortality method and the *Rspan* method. The mortality method (1/adult mortality + age of first reproduction) is only practicable if the annual mortality after the age of first reproduction is well known, and if mortality and fecundity do not change with age after the age of first reproduction (i.e., there is no reproductive senescence) [9]. The *Rspan* method is a useful approximation to the life-table method when ages of first and last reproduction are the only available data, which is often the case, especially for lesser-known taxa. So we employed the *Rspan* method across our focal group due to its high applicability to the focal species (few species had life-table or annual mortality data) and to other taxa in general. The *Rspan* approach was also recently found to be largely unbiased on average [8]. Generation length was therefore calculated as follows: Generation length = Rspan * z + AFR where *Rspan* is the species reproductive life span, calculated as the difference between the age at last reproduction (ALR) and the age of first reproduction (AFR), and z is a constant “depending on survivorship and relative fecundity of young versus old individuals in the population” [9]. That is, z is the fraction of adult life span that passes before the average individual has contributed half of its lifetime, age-weighted reproduction [8].

To implement the *Rspan* method we compiled a database (S1 Data) for the focal taxa. First, we devised a set of simple rules, such as when to use the median or maximum values, to ensure consistent estimation of relevant life-history parameters from multiple sources. These parameters included age of first reproduction (AFR), age of last reproduction in the wild (ALRw), age of last reproduction in captivity (ALRc) and adult body-mass. We obtained data from five main sources [13–17] for all parameters except for adult body-mass, which we sourced from the PanTHERIA database [18].

#### Age at first reproduction (AFR)

Age at first reproduction (AFR) is defined as the age at which individuals first produce offspring in the wild (which may be later than when they are biologically capable of breeding), averaged over all breeding individuals [9]. We used empirical estimates of AFR averaged over males and females when they were available; however for the majority of species we summed estimates of age at female sexual maturity and gestation length. Where range estimates were given, e.g. 6–8 months, we took the central value. We then calculated the median across the available AFR estimates from the literature (1 ≥ n ≤5; mean n = 3.1 sources) and rounded up to the nearest day [3]. We purposefully did not base any estimates solely on the age of sexual maturity for males (which have been used previously [19]), as this does not account for the inherent time periods and processes (e.g., successful breeding, gestation length) that dictate the timing of first breeding and thus contribute to a species generation length under the IUCN definition.

#### Age at last reproduction (ALR)

We found empirical estimates of ALR for only four species, so for the remaining species we used maximum longevity as a proxy for ALR and assumed no reproductive senescence, following the IUCN Standards and Petitions Subcommittee [9]. We then took the maximum empirical estimate for longevity across all data sources and where we obtained range estimates, e.g. 10–12 years, we selected the upper bound (to avoid bias from life expectancy estimates). There is potential for bias in *Rspan* estimates calculated from maximum longevity based on captive individuals, which has previously been assumed to only influence a limited number of large-bodied species [19]. Yet here, we recognize this bias as a significant issue, as large species are more likely to be assessed based on a multiple-generation length time horizon than a fixed time period, due to the scaling of generation length with body-mass (S1 Appendix). So here we separated estimates of age of last reproduction into captive (ALRc) and wild (ALRw) values. This is often difficult with the majority of sources lacking specificity on the type of data. So, following a precautionary approach, we only recorded data as wild if the source clearly stated it was based on wild individuals (1 ≥ n ≤5; mean n = 2.0 sources); otherwise we assumed it to be captive (1 ≥ n ≤5; mean n = 2.6 sources).

#### Generation length

Using the values of AFR, ALRw and ALRc explained above we calculated estimates of generation length in the wild (GLw) for 54 species using the *Rspan* method and, for comparison, captive estimates (GLc) for 64 species. We used a previously quantified (based on 221 species) value of 0.29 for z [19]. We therefore maintain consistency across mammalian species and facilitate comparison between methods.

### Missing-data species

#### Model testing

Each model included all complete cases (i.e., excluding those with missing-data) resulting in a variable number of species used in each test. All statistical analyses were performed in R, version 3.3.2 [20], using functions from the MPSEM package [21] unless stated otherwise.

To test the explanatory power of phylogenetic and allometric factors as predictors of generation length we employed Phylogenetic Eigenvector Maps (PEMs). PEMs use a matrix containing the graphic structure of a phylogeny to calculate eigenvectors that are later used as descriptors (predictors) in predictive modeling procedures such as multiple regression [22].

Here, we used a phylogenetic supertree (with best estimates of divergence times) of extant mammals [23], with updates [12]. We first dropped non-focal species (i.e., non-antelopes) from the tree (leaving 86 species), then converted this tree to a phylogenetic graph using the *Phylo2DirectedGraph* function and built a PEM from it using the *PEM*.*build* function. We optimized the steepness parameter—describing whether changes occur, on average, progressively along edges or abruptly at vertices [21]—for the phylogeny by implementing *PEM*.*fitSimple*, with GLw and log_10_ body-mass as the auxiliary traits. The function *PEM*.*fitSimple* performs a maximum likelihood estimation of the steepness parameter assuming single values for the whole tree [21]. We then performed stepwise variable addition in a multiple regression framework using the *lmforwardsequentialAICc* function; with 85 phylogenetic eigenvectors (n-1) and log_10_ adult body-mass as predictors, and GLw as the response variable. Early eigenvectors (closer to 1) represent divergences closer to the root of the phylogeny and later eigenvectors (closer to 85) represent the fine-scale differences among species [24]. Model selection was based on Akaike’s Information Criterion corrected for small sample size (AICc) [25].

We subsequently calculated the relative importance of the predictors, using the *calc*.*relimp* function (relaimpo package) [26]. We employed the Genizi metric [26,27], which decomposes R^2^ into components that serve as descriptive intuitive statistics indicating the relative importance of each regressor with respect to its overall effect on the dependent variable [27].

Like all phylogenetic methods, the results of the PEM are dependent on the topology and branch lengths of the phylogeny. To test the uncertainty of the phylogeny we repeated the analyses with the lower and upper confidence intervals for the divergence times [12,23], and with all branch lengths set to the same length (reflecting the scenario of the lack of a well-established phylogeny, as for many non-mammalian clades) (S2 Appendix).

#### Testing prediction frameworks

After identifying the best explanatory variables for generation length we employed these in two predictive frameworks: 1) predictive PEM; and 2) binning approaches split into a) taxonomic ranks and body-mass, and b) taxonomic rank. We then used leave-one-out cross-validation to compare the predicted values of generation length to observed values and therefore assess the accuracy of each framework. Leave-one out cross-validation is an estimate of the generalization performance of a model trained on n-1 samples.

The first predictive approach was based on the previously used PEM, where we used the *getGraphLocations* function to identify the simulated missing species and then repeated the optimization and model selection process previously described (i.e., implementing the *PEM*.*fitSimple* and *lmforwardsequentialAICc* functions) to produce a best fitting model. We then used the *predict* function to calculate generation length estimates from the selected model with 95% confidence intervals.

Second we evaluated two simple binning approaches: (a) using taxonomic ranks (genus/family) together with binned log_10_ body-mass [19]; and (b) using taxonomic rank alone [9,19,28]. We implemented both these techniques on our dataset, calculating the mean generation length of congenerics or confamilials in the same bin of log_10_ body-mass and the mean generation length of congenerics or confamilials irrespective of their body-mass for each removed species under a leave-one-out cross-validation routine.

We summarized the strength of the PEM and binning approaches by using prediction coefficients (P^2^) [22], whereby *P*^2^ closer to 1 indicates perfect predictability. As well as testing the fit of the predictions against the null hypothesis of slope = 1 and intercept = 0 (i.e., no difference between the observed and predicted values).

#### Missing-data species

We also used the PEM and binning approaches to calculate generation length for true missing-data species (i.e., those species that did not have the required life-history information needed to calculate GLw directly; n = 32 species). To implement the PEM approach we ran the *getGraphLocations* function to obtain the locations of the missing-data species on the phylogenetic tree. This information, plus the selected model from earlier testing, was supplied to the *predict* function to calculate estimates of generation length with 95% confidence intervals.

We also used both binning approaches combined to predict generation length for the missing-data species. We had to use both combined as not every species had a reference species in the same bin of log_10_ body-mass to base the estimation on. To integrate the two binning methodologies we used a hierarchical selection process, where the order was based on the findings from earlier model testing. First priority was any estimate based on congenerics in the same bin of body-mass, if this was not available then an estimate from confamilials in the same body-mass bin was used, then congenerics irrespective of body-mass and finally confamilials irrespective of body-mass.

## Results

### Age at last reproduction (ALR)

**Fig 2:**
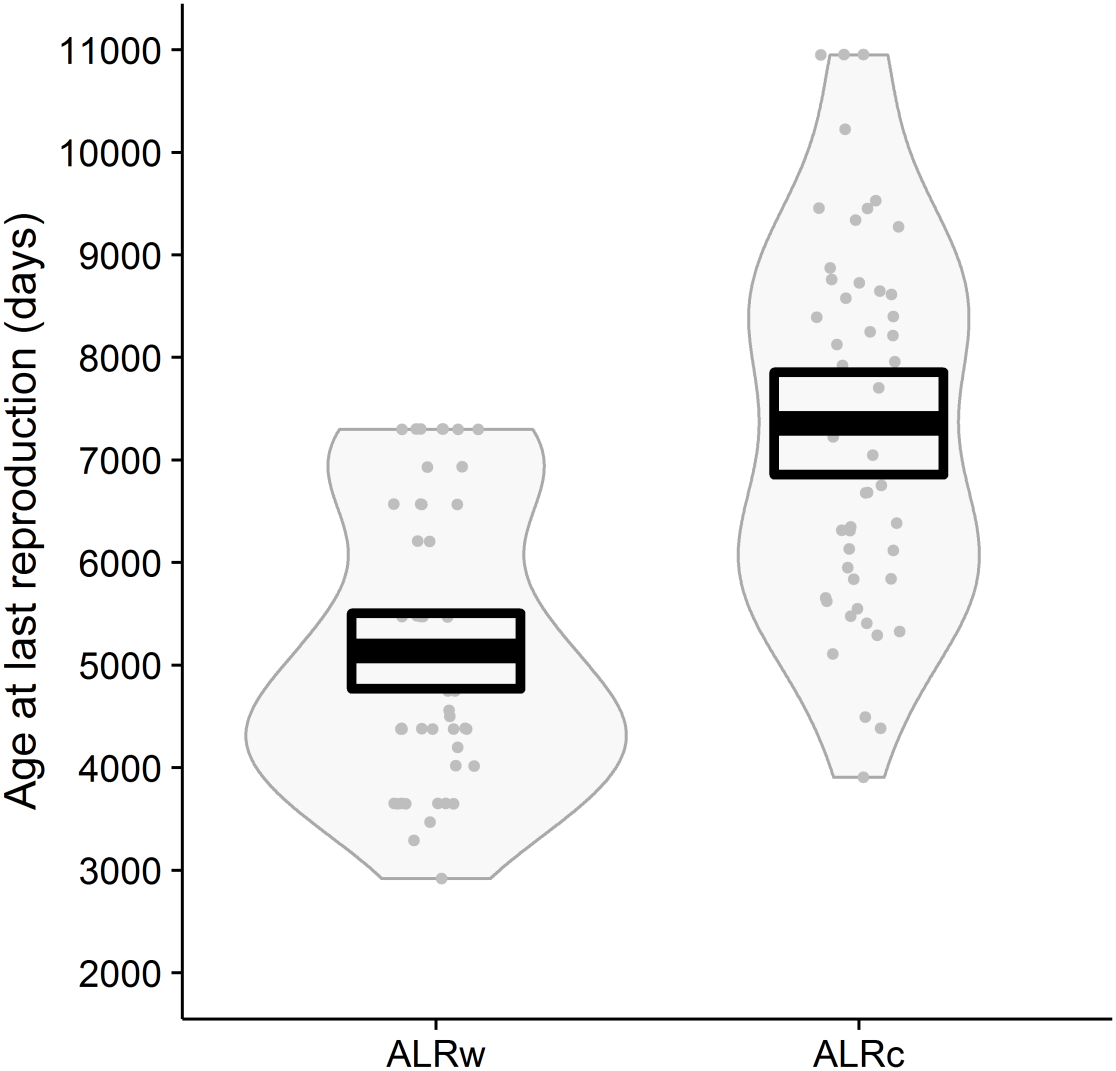
An RDI (Raw data, descriptive statistic and inferential statistic) plot of age at last reproduction in the wild (ALRw) and age at last reproduction in captivity (ALRc)—for 50 species (excluding 36 species with missing data). The plot includes the mean as a thick black line, 95% confidence intervals as a black rectangle, a grey bean plot of data density and grey points of jittered data.

We found that estimates of ALRc were higher, mean of the differences = 2218 days (95% confidence interval 341 days), than ALRw, t_49_ = 13.08, *p* < 0.001, confirming that captive and wild estimates of age at last reproduction are nonequivalent and justifying the careful separation of longevity estimates (Fig 2). These differences in ALR sum through to a mean difference of 1.8 years (95% confidence interval 0.3 years) between GLw and GLc.

### Model testing

Generation length was best explained (lowest AICc) by log_10_ body-mass and 18 phylogenetic eigenvectors (of the 85 phylogenetic eigenvectors available), with body-mass explaining 64% and phylogeny (summed relative importance of the 18 phylogenetic eigenvectors) 36% of the partitioned explained variance (S2 Appendix). The most important phylogenetic eigenvectors were 12 and 8, which explained 8% and 5% of the partitioned explained variance respectively, suggesting that divergences closer to the root of the phylogeny were more important (S2 Appendix).

### Testing prediction frameworks

**Fig 3:**
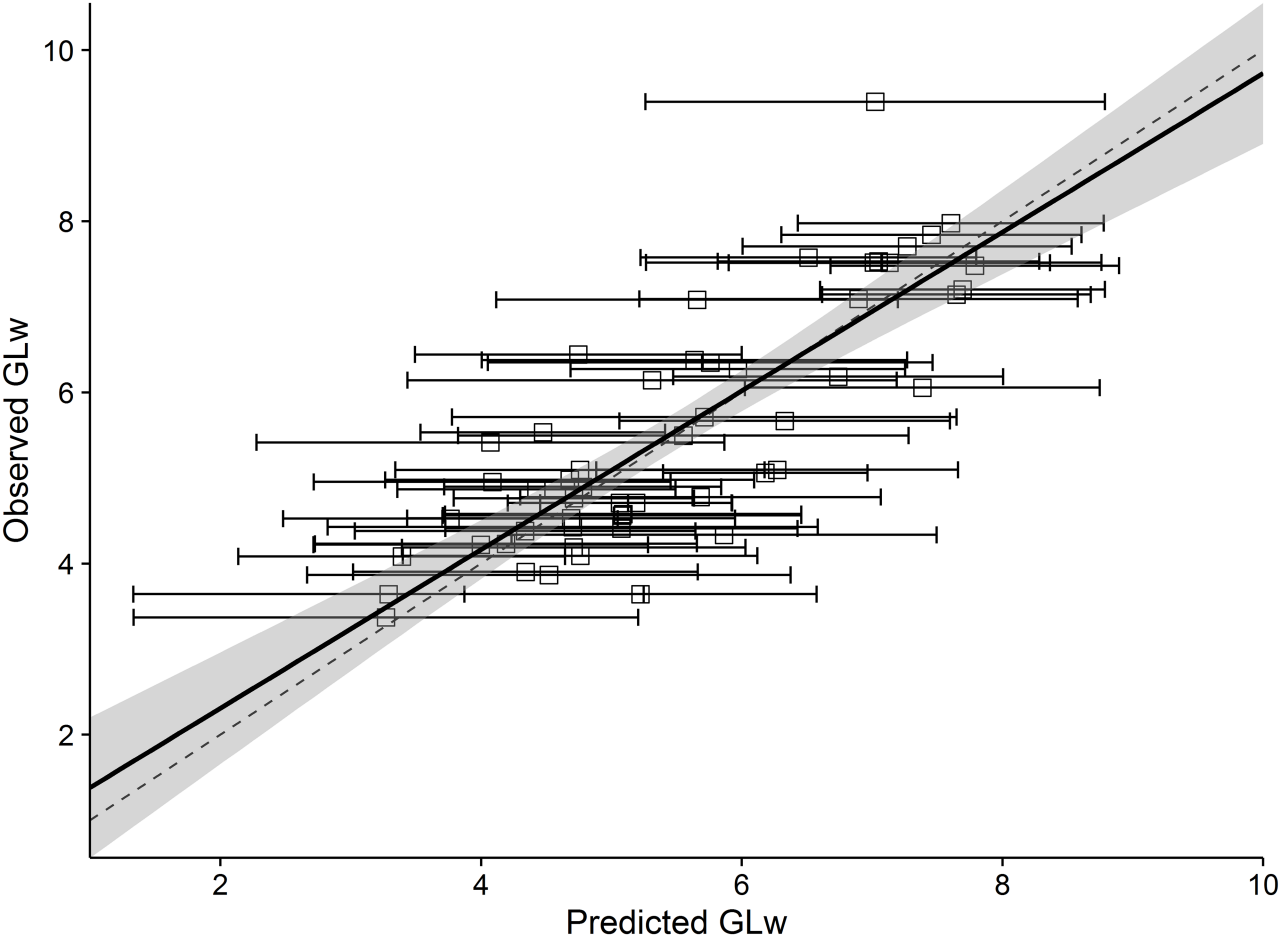
Observed and PEM (Phylogenetic eigenvector map) predicted values of generation length in the wild (GLw) obtained following leave-one-out cross-validation—for 54 species (excluding 32 species with missing data). The dashed line is a 1:1 line, the solid black line is a regression line of observed values as a function of predictions, and the grey envelope represents the 95% confidence limits of the regression line. Horizontal bars are limits of the 95% confidence intervals.

The PEM predicted values of GLw showed congruence with the observed values of GLw (Fig 3) and the model was reasonably accurate, with a *P*^*2*^ of 0.68. The regression between the observed and predicted values did not differ from the null hypothesis of slope = 1 and intercept = 0 (slope = 0.93, *p* = 0.41 and intercept = 0.46, *p* = 0.36). Therefore the disagreement between the PEM predictions and observed data is due entirely to the unexplained variance and not because of inconsistencies or bias.

**Fig 4:**
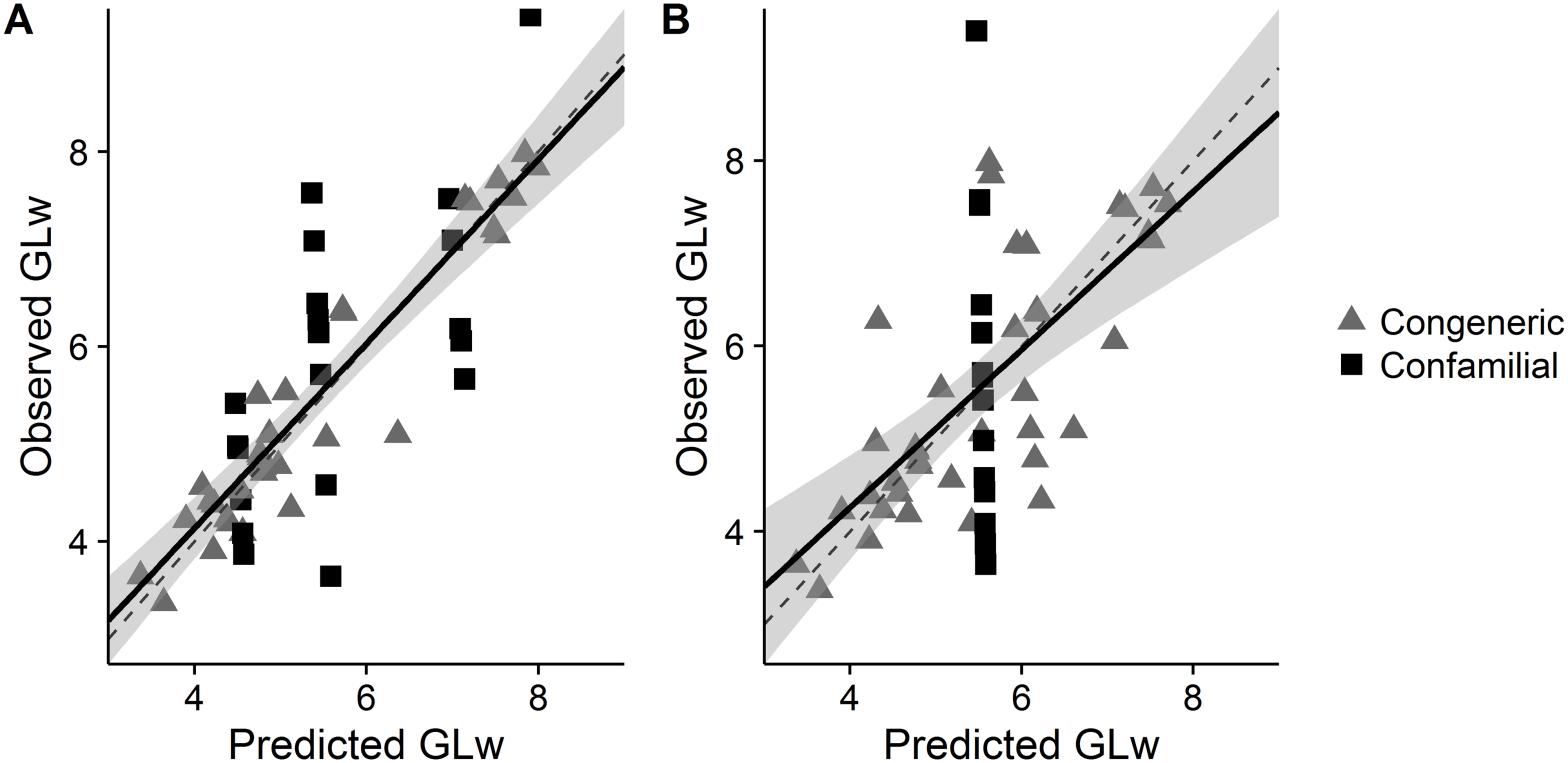
Observed and binning approach predicted values of generation length in the wild (GLw) obtained following leave-one-out cross-validation—for 54 species (excluding 32 species with missing data). Predictions are presented when incorporating bins of log_10_ body-mass (A) and irrespective of body-mass (B). The dashed lines are 1:1 lines, the solid black lines are regression lines of the observed values as a function of predictions and the grey envelopes represent the 95% confidence limits of the regression lines.

For the first binning approach (including body-mass), 34 species were assigned the mean GLw from congeneric species in the same bin of log_10_ body-mass and 20 species from confamilial species in the same bin of body-mass (Fig 4A). The prediction coefficient was 0.73 and therefore the binning approach is reasonably accurate when including body-mass. The regression did not differ from the null hypothesis (slope = 0.95, *p* = 0.51 and intercept = 0.35, *p* = 0.44).

For the second binning approach (ignoring body-mass), 40 species were assigned the mean GLw from congeneric species and 14 species from confamilial species (Fig 4B). Accuracy was poor when we excluded body-mass from the binning process, with a *P*^*2*^ of 0.37. Although the regression did not differ from the null hypothesis (slope = 0.85, *p* = 0.34 and intercept = 0.85, *p* = 0.33).

All approaches (PEM, binning including body-mass and binning excluding body-mass; Figs 3 and 4) tended to overestimate GLw for low observed values and underestimate GLw for high observed values.

### Missing-data species

There was agreement between the PEM approach and the combined binning approach for the true missing-data species, mean of the differences = -0.036 years (95% confidence interval 0.25 years), t_31_ = -0.29, *p* = 0.77.

All findings were similar when we excluded chiru and African buffalo (S3 Appendix). In addition, all results based on the PEM approach were qualitatively similar for the lower and upper date estimates, and when all branch lengths were set to equal length (S2 Appendix; S1 Data).

## Discussion

Here we present a transparent, consistent and transferable workflow for improving the use of the *Rspan* method for calculating generation length. We have obtained more accurate values of generation length across a suite of ungulates for use in the Red List, by taking care to separate data from wild and captive sources and enhancing predictions for missing-data species (implementing a PEM/better accounting for body-mass). There are however inevitable caveats concerning our analysis, but we believe that some of the principal potential biases that might affect the *Rspan* method have been minimized and appropriate approximations have been suggested where data are lacking. Both of these issues could be reduced further primarily by the inclusion of additional data. For example the moderate prediction coefficients (for the PEM and binning approaches) demonstrate that even good proxies could be improved by having the necessary data to calculate generation length directly [8]. Moreover, here the workflow has been applied to well studied taxa, but the process may have lower accuracy for taxonomic groups with less primary data; although in this situation, there would likely be more missing-data species and a robust methodology to predict GL may be increasingly valuable.

We have shown that captive and wild data, when considering ALR, are nonequivalent (Fig 2). We therefore suggest that the introduction of bias from mixing wild and captive life-history data may be a widespread but under-acknowledged problem in conservation biology. So here we recommend explicitly stating the type of data used and separating captive and wild data where possible. However differentiating between captive and wild data may be problematic for less well studied taxa (e.g., where no wild data exist or where the type of data presented by the source is unclear). In these instances we promote the use of multiple data sources and PEMs to approximate generation length, rather than incorporating substantial amounts of captive data. But at the very least we advise that the biological implications of mixing both wild and captive data are considered in life-history analyses.

Extrapolation of generation length for missing-data species is essential for lesser-known and potentially threatened taxa [9]. We have shown statistically that both allometry (adult body-mass) and phylogeny (phylogenetic eigenvectors and taxonomic ranks) are important predictors of generation length (S2 Appendix) and should be used in combination to extrapolate generation length, as has been coarsely applied previously for calculating generation length for antelopes (in 2008) and other taxa, but was previously untested. We show the unequivocal importance of both allometry and phylogeny, while also deriving scaling relationships (S1 Appendix) and predictive frameworks (Figs 3 and 4) that allow generation length to be estimated more accurately for missing-data species and for outliers in the existing data to be identified. For example, the generation lengths for three duiker species: Ader’s duiker (*Cephalophus adersi*), white-bellied duiker (*Cephalophus leucogaster*) and Abbott’s duiker (*Cephalophus spadix*), were previously estimated at 1.9, 2.3 and 2.6 respectively [19], whereas we produced estimates of 4.2, 4.6 and 6.0, based on PEMs, which are more consistent with others in the genus *Cephalophus* and for antelope of similar size.

To obtain estimates for missing-data species we recommend the use of PEMs to predict generation length, as they are approximately as accurate as the binning approach that included body-mass, but are less dependent on species having available reference species (i.e., comparison species in the same genus/family/body-mass bin) and more explicitly account for phylogenetic relatedness. However, as with all phylogenetic methods, PEMs are subject to phylogenetic uncertainty. Here we have shown that the PEM approach is robust to the phylogenetic hypothesis and to the lack of a well-established phylogeny (S2 Appendix), giving the technique a broad taxonomic scope. We have made a tutorial version of the R code for implementing the PEMs available (S1 R script). However where PEMs are not applicable or feasible (e.g., where phylogenetic trees are not available) the approach of assigning the mean generation length of congeneric or confamilial species in the same bin of log_10_ body-mass is justifiable (R code for the binning approach is also available [S2 R script] or can be implemented manually in Microsoft Excel [not provided]). Yet we caution against the extrapolation of generation length based solely on taxonomy, because body-mass was shown to be important statistically (and biologically). In fact the relative importance of body-mass was shown to be almost twice that of phylogeny and therefore we believe allometric data should always be included when predicting generation length. The findings reported here, however, may require further investigation across a variety of taxonomic groups and body sizes to establish their generality. Although, linear relationships have previously been reported between generation length and log body mass for birds [29], suggesting these associations may hold for other taxa.

**Fig 5:**
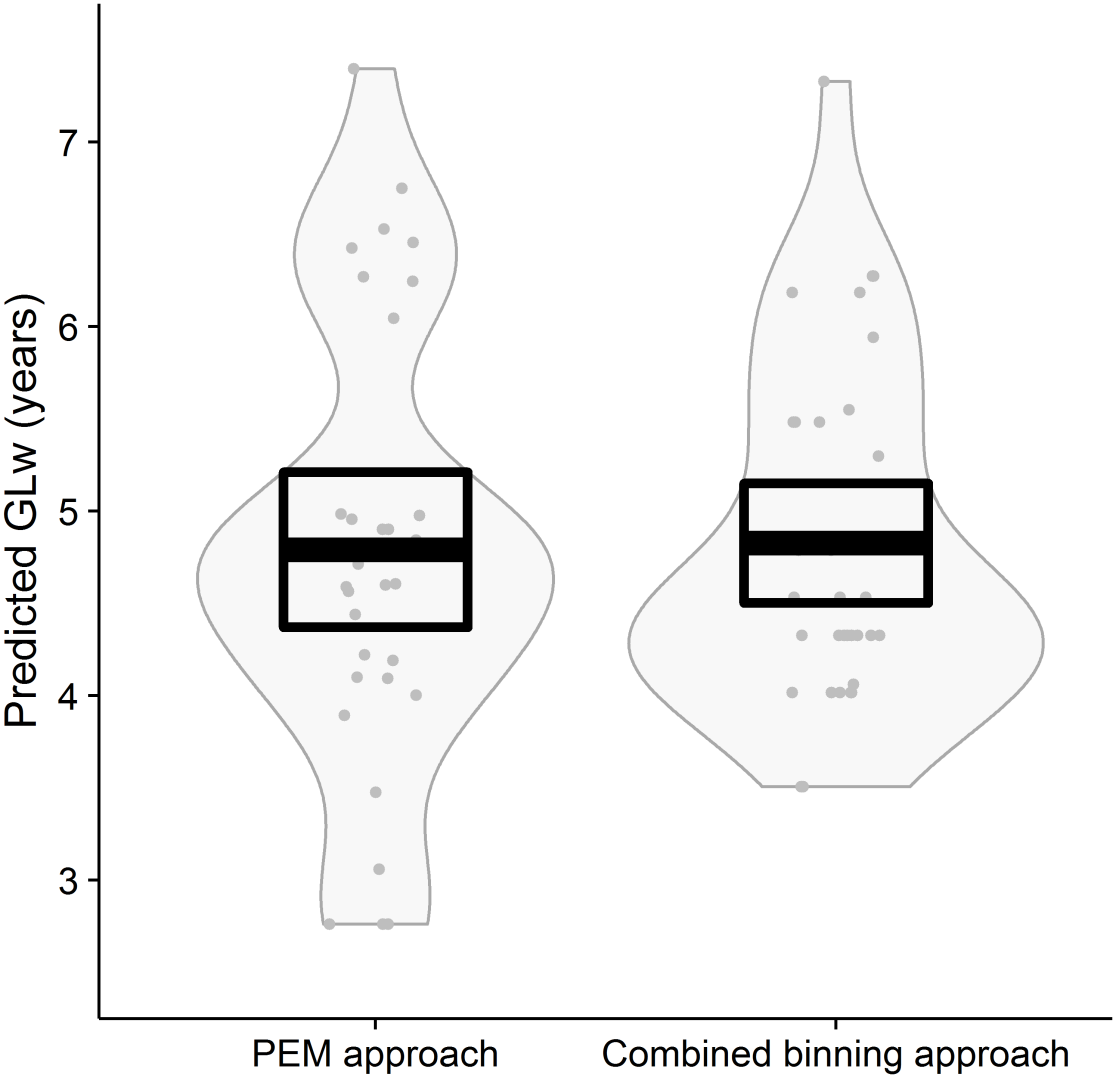
A comparison of predicted generation length in the wild (GLw) following a Phylogenetic Eigenvector Map (PEM) approach and a combined binning approach—for 32 true missing-data species. The plot includes the mean as a thick black line, confidence intervals as a black rectangle, a grey bean plot of data density and grey points of jittered data.

Implementing our PEM workflow enabled us to estimate generation length for all 86 extant species (including 32 missing-data species; Fig 5) that fall within the IUCN SSC Antelope Specialist Group’s remit and then apply these values in the updated Red List (some values used in the 2016 assessments were preliminary results), for example Tibetan gazelle (*Procapra picticaudata*) [30]. Finally, while we have shown that predictions for generation length can be considerably improved using our approach, accurate estimates of generation length in the end depend on species-specific life-history data and every effort should be made to collect these from wild populations.

## Supporting information

S1 Appendix Regression of generation length in the wild on body-mass. (DOCX)

S2 Appendix Phylogenetic uncertainty analyses, including the relative importance of predictors for generation length under a Phylogenetic Eigenvector Map approach. (DOCX)

S3 Appendix Phylogenetic Eigenvector Map and binning approaches when excluding chiru (*Pantholops hodgsonii*) and African buffalo (*Syncerus caffer*). (DOCX)

S1 Data Collated life-history data for antelope, including generation length estimates under different phylogenetic hypotheses. (XLSX)

S2 Data Tutorial life-history data for antelope, provided for use in R tutorials. (CSV)

S1 R script R tutorial for the Phylogenetic Eigenvector Map approach. (R)

S2 R script R tutorial for the binning approach. (R)
