# Supplementary material for: Improving generation length estimates for the IUCN Red List
Source: PLoS One. 2018 Jan 25;13(1):e0191770. doi: 10.1371/journal.pone.0191770 (PMC5784970; doi:10.1371/journal.pone.0191770)
Supplement: S1 Appendix — (DOCX) [file pone.0191770.s001.docx]

S1 Appendix. Regression of generation length in the wild on body-mass

When assessing the relationship between body-mass and GLw under a univariate regression analysis we found that GLw increased linearly with increasing log_10_ body-mass, R^2^ = 0.68, F_1,52_ = 111.4, *p* < 0.001 (Fig 1).


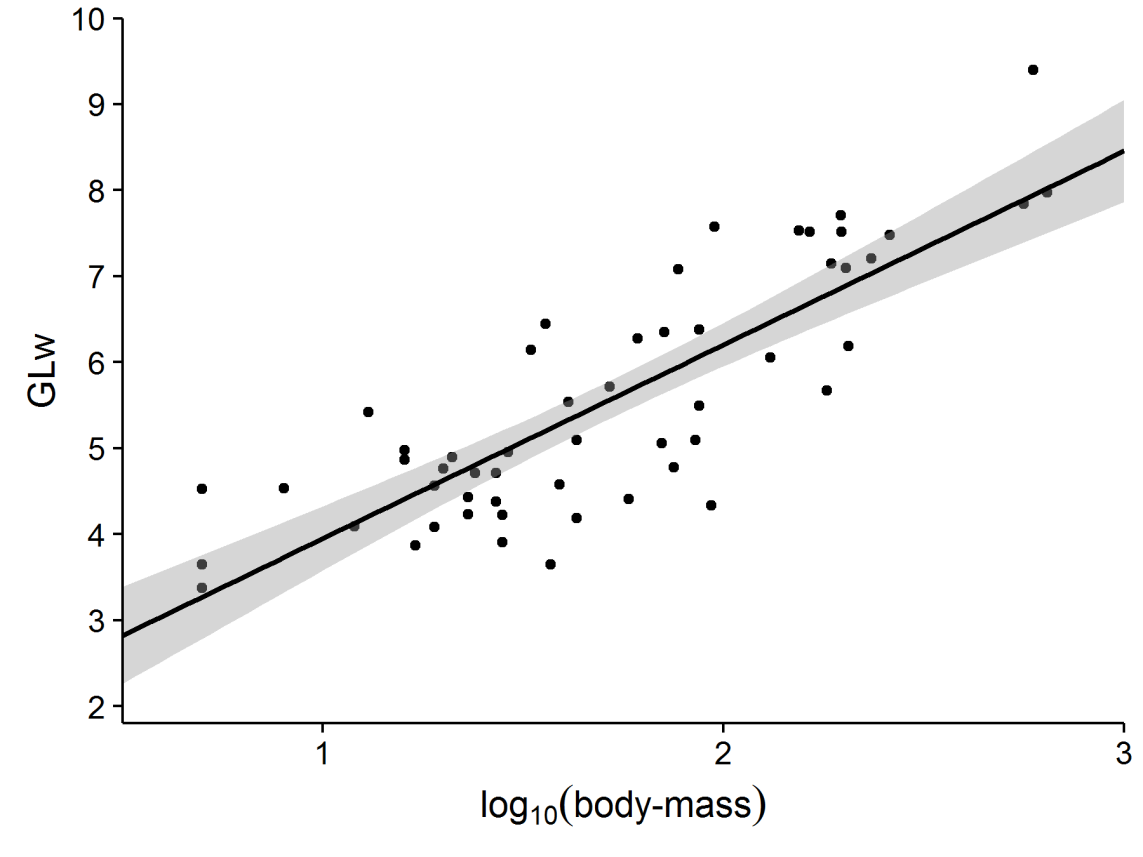


**Fig 1. Generation length in the wild (GLw) as a function of log_10_ body-mass.** The 95% confidence limits of the regression line (solid black line) are represented by a grey envelope.
