# Supplementary material for: Improving generation length estimates for the IUCN Red List
Source: PLoS One. 2018 Jan 25;13(1):e0191770. doi: 10.1371/journal.pone.0191770 (PMC5784970; doi:10.1371/journal.pone.0191770)
Supplement: S2 Appendix — (DOCX) [file pone.0191770.s002.docx]

S2 Appendix. Phylogenetic uncertainty analyses

Here we test the Phylogenetic Eigenvector Map approach under different phylogenetic hypotheses: best divergence time estimates, lower CI for divergence time estimates, upper CI for divergence time estimates, and when all branches are set to equal length (Tables 1 and 2).

We subsequently calculated the relative importance of the predictors (Table 1), using the *calc.relimp* function (relaimpo package) [26]. We employed the Genizi metric [27], which decomposes R^2^ into components that serve as descriptive intuitive statistics indicating the relative importance of each regressor with respect to its overall effect on the dependent variable [26,27].

Although the composition of the phylogenetic eigenvectors differed between phylogenetic hypotheses, the contribution of body mass (49-64%) and phylogeny (summed relative importance: 36-51%) to the best fitting models was similar (Table 1), and phylogenetic eigenvectors closer to the root of the phylogeny were generally more important (Table 1). Moreover the ability of the PEMs to predict the observed data was similar (P^2^ 0.68-0.73) across the phylogenetic hypotheses (Table 2), and even when using a limited phylogeny (equal branch length), suggesting a broad taxonomic scope for the approach (i.e., applicable to taxonomic groups without well-established phylogenies).

**Table 1. The relative importance of the predictors included in the best fitting model for a Phylogenetic Eigenvector Map approach under different phylogenetic hypotheses (best divergence times [Best], lower CI for divergence times [Lower], upper CI for divergence times [Upper], and when all branches are set to equal length [Equal]).**

| Predictor | Relative importance | | | |
| --- | --- | --- | --- | --- |
|  | Best | Lower | Upper | Equal |
| Log10(body-mass) | 0.64 | 0.59 | 0.51 | 0.49 |
| E1* |  |  | 0.05 |  |
| E2 | 0.04 | 0.04 | 0.03 | 0.06 |
| E3 |  |  | 0.13 | 0.09 |
| E4 | 0.03 | 0.04 | 0.02 |  |
| E5 |  | <0.01 | <0.01 |  |
| E6 |  |  |  | 0.06 |
| E8 | 0.05 | 0.06 | 0.03 |  |
| E9 |  | 0.02 | 0.02 | 0.01 |
| E10 | 0.02 | 0.05 |  | <0.01 |
| E11 | 0.01 | 0.10 | <0.01 |  |
| E12 | 0.08 |  |  | 0.03 |
| E13 | 0.01 |  | 0.01 |  |
| E14 | 0.01 |  |  | 0.01 |
| E15 |  | 0.01 |  |  |
| E16 |  |  | 0.05 |  |
| E17 |  |  | 0.02 | 0.01 |
| E18 |  |  | <0.01 |  |
| E19 | 0.01 | 0.01 | 0.01 | 0.04 |
| E20 |  |  | 0.01 | 0.08 |
| E21 | 0.03 | 0.01 | 0.02 | 0.03 |
| E22 | 0.01 |  | <0.01 | 0.02 |
| E23 | 0.01 | 0.01 | <0.01 | 0.01 |
| E24 |  |  | 0.01 |  |
| E25 |  | 0.01 | 0.01 |  |
| E26 |  | 0.01 |  | <0.01 |
| E27 |  |  |  | 0.01 |
| E29 | 0.01 |  |  |  |
| E30 | 0.01 | 0.01 | 0.01 |  |
| E31 | 0.01 |  | 0.01 |  |
| E32 |  |  | 0.01 |  |
| E33 |  | 0.01 | 0.01 |  |
| E35 | 0.03 |  | <0.01 | <0.01 |
| E36 |  |  | <0.01 |  |
| E37 |  |  | 0.01 | 0.01 |
| E38 | <0.01 | 0.02 |  |  |
| E39 |  | 0.02 |  |  |
| E40 |  |  | <0.01 |  |
| E41 |  |  | 0.02 |  |
| E42 |  |  |  | 0.01 |
| E44 |  |  |  | 0.01 |
| E46 |  |  |  | <0.01 |
| E47 |  | <0.01 |  |  |
| E48 |  |  | <0.01 | 0.01 |
| E50 |  |  |  | <0.01 |
| E51 | <0.01 |  | <0.01 | <0.01 |
| * E represents phylogenetic eigenvector | | | | |

**Table 2. Testing the Phylogenetic Eigenvector Map approach predictive power under different phylogenetic hypotheses (best divergence times [Best], lower CI for divergence times [Lower], upper CI for divergence times [Upper], and when all branches are set to equal length [Equal]).** Statistics include the prediction coefficient (P^2^) and the slope and intercept of the regression between the observed and predicted values of generation length in the wild, for each phylogenetic hypothesis.

| Statistic | Phylogenetic sensitivity test | | | |
| --- | --- | --- | --- | --- |
|  | Best | Lower | Upper | Equal |
| P^2^ | 0.68 | 0.73 | 0.71 | 0.68 |
| Slope | 0.93 (*p* = 0.41) | 0.92 (*p* = 0.29) | 0.89 (*p* = 0.19) | 0.92 (*p* = 0.38) |
| Intercept | 0.46 (*p* = 0.36) | 0.50 (*p* = 0.27) | 0.66 (*p* = 0.14) | 0.43 (*p* = 0.40) |
