# Supplementary material for: Improving generation length estimates for the IUCN Red List
Source: PLoS One. 2018 Jan 25;13(1):e0191770. doi: 10.1371/journal.pone.0191770 (PMC5784970; doi:10.1371/journal.pone.0191770)
Supplement: S3 Appendix — (DOCX) [file pone.0191770.s003.docx]

S3 Appendix. Analyses when excluding chiru and African buffalo

Phylogenetic eigenvector map approach excluding chiru and African buffalo

When excluding chiru and African buffalo, the PEM predicted values of GLw showed congruence with the observed values of GLw (Fig 1), and the model was reasonably accurate, with a *P*^2^ of 0.70. The regression did not differ from the null hypothesis (slope = 0.86, *p* = 0.08 and intercept = 0.76, *p* = 0.09).


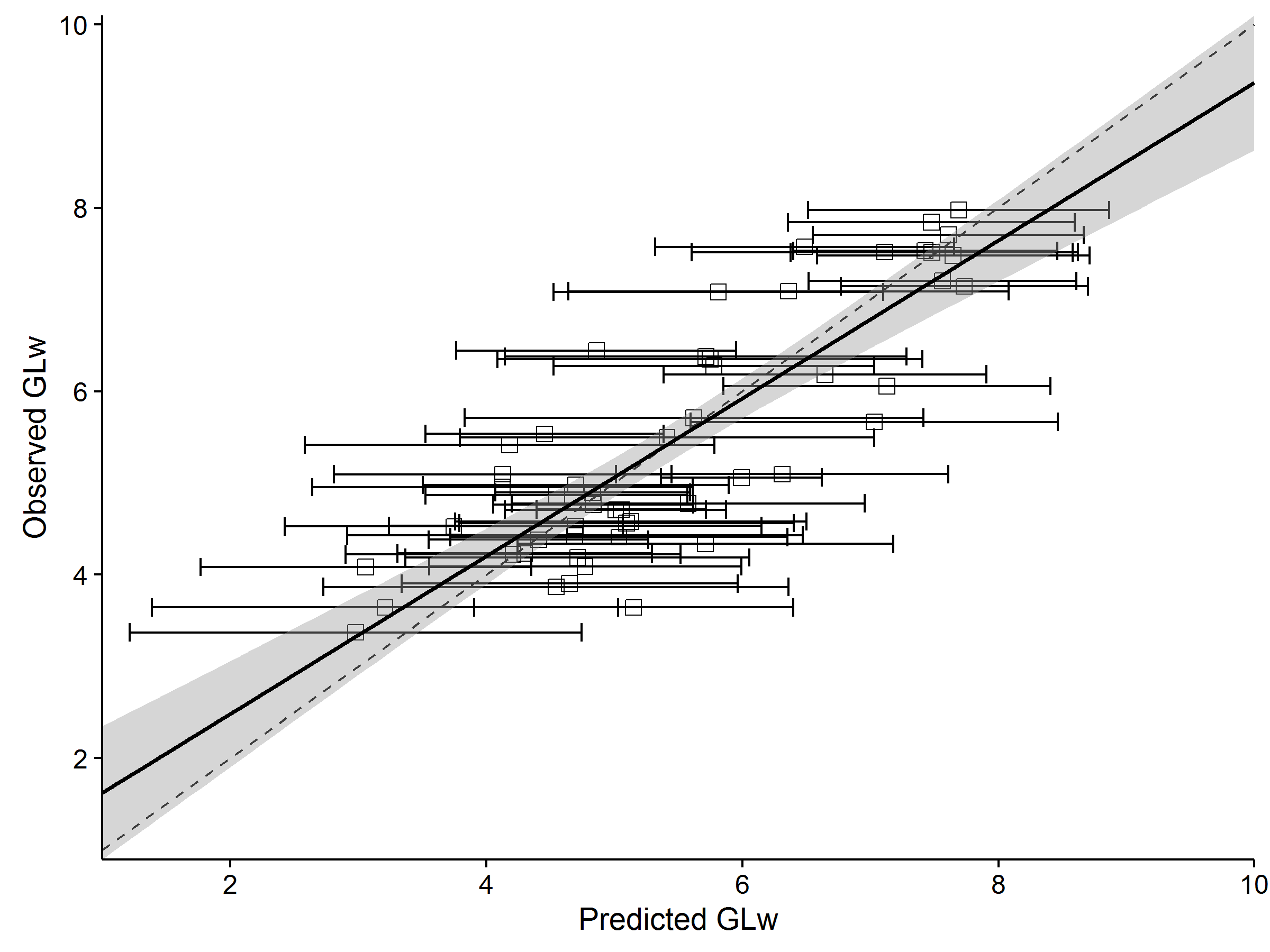


**Fig 1. Observed and PEM (Phylogenetic Eigenvector Map) predicted values of generation length in the wild (GLw) obtained following leave-one-out cross-validation - for 52 species (excluding 32 species with missing data and chiru (*Pantholops hodgsonii*) and African buffalo (*Syncerus caffer*)).** The dashed line is a 1:1 line, the solid black line is a regression line of observed values as a function of predictions, and the grey envelope represents the 95% confidence limits of the regression line. Horizontal bars are limits of the 95% confidence intervals.

Binning approach excluding chiru and African buffalo

When excluding chiru and African buffalo, the prediction coefficient for the binning approach including body mass was 0.71 (Fig 2A). The regression did not differ from the null hypothesis of slope = 1 and intercept = 0 (slope = 0.90, *p* = 0.20 and intercept = 0.59, *p* = 0.20).

The prediction coefficient for the binning approach when excluding body mass was 0.44 (Fig 2B). The regression did not differ from the null hypothesis (slope = 0.86, *p* = 0.30 and intercept = 0.75, *p* = 0.33).


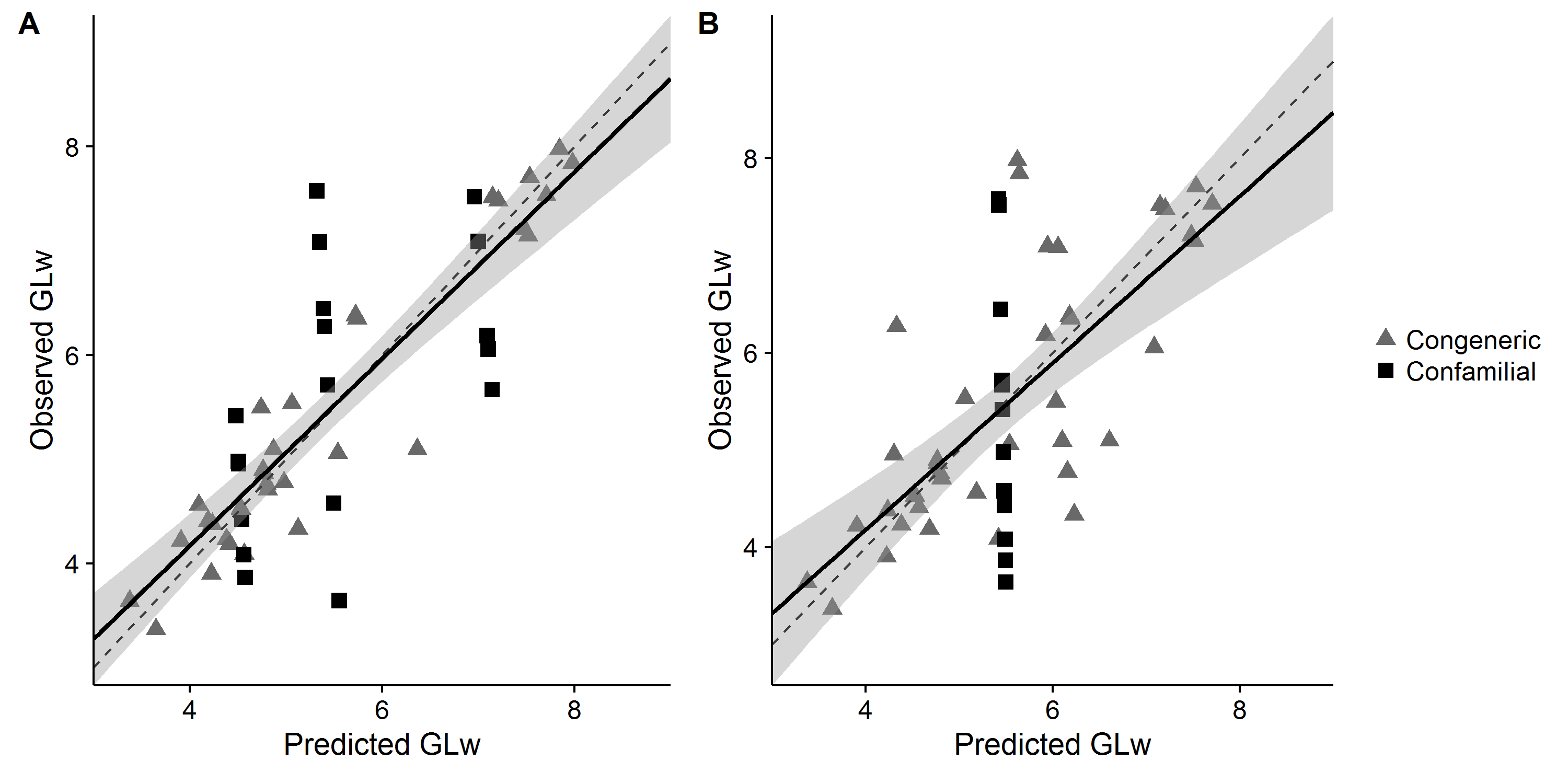


**Fig 2. Observed and binning approach predicted values of generation length in the wild (GLw) obtained following leave-one-out cross-validation - for 52 species (excluding 32 species with missing data and chiru (*Pantholops hodgsonii*) and African buffalo (*Syncerus caffer*)).** Predictions are presented when incorporating bins of log_10_ body-mass (A) and irrespective of body-mass (B). The dashed lines are 1:1 lines, the solid black lines are regression lines of the observed values as a function of predictions and the grey envelopes represents the 95% confidence limits of the regression lines.

Relative importance of predictors excluding chiru and African buffalo

**Table 1. The relative importance of the predictors included in the best fitting model when excluding chiru (*Pantholops hodgsonii*) and African buffalo (*Syncerus caffer*).**

| Predictor | Relative importance | |
| --- | --- | --- |
|  | Best | Excluding chiru and buffalo |
| Log10(body-mass) | 0.64 | 0.59 |
| E1* |  | 0.05 |
| E2 | 0.04 | 0.05 |
| E4 | 0.03 | 0.03 |
| E7 |  | 0.09 |
| E8 | 0.05 |  |
| E10 | 0.02 | 0.02 |
| E11 | 0.01 |  |
| E12 | 0.08 | 0.01 |
| E13 | 0.01 | 0.01 |
| E14 | 0.01 | 0.01 |
| E16 |  | 0.01 |
| E18 |  | 0.01 |
| E19 | 0.01 |  |
| E20 |  | 0.02 |
| E21 | 0.03 | <0.01 |
| E22 | 0.01 |  |
| E23 | 0.01 | 0.01 |
| E27 |  | 0.01 |
| E28 |  | 0.01 |
| E29 | 0.01 | 0.01 |
| E30 | 0.01 | 0.01 |
| E31 | 0.01 |  |
| E32 |  | <0.01 |
| E33 |  | 0.03 |
| E35 | 0.03 | <0.01 |
| E38 | <0.01 |  |
| E40 |  | 0.01 |
| E49 |  | <0.01 |
| E51 | <0.01 |  |
| * E represents phylogenetic eigenvector | | |
